# Association mapping for water use efficiency in soybean identifies previously reported and novel loci and permits genomic prediction

**DOI:** 10.3389/fpls.2024.1486736

**Published:** 2024-11-28

**Authors:** Siva K. Chamarthi, Larry C. Purcell, Felix B. Fritschi, Jeffery D. Ray, James R. Smith, Avjinder S. Kaler, C. Andy King, Jason D. Gillman

**Affiliations:** ^1^ Division of Plant Science & Technology, University of Missouri, Columbia, MO, United States; ^2^ Department of Crop, Soil, and Environmental Sciences, University of Arkansas, Fayetteville, AR, United States; ^3^ Crop Genetics Research Unit, United States Department of Agriculture – Agricultural Research Service (USDA-ARS), Stoneville, MS, United States; ^4^ Plant Genetic Research Unit, United States Department of Agriculture – Agricultural Research Service (USDA-ARS), University of Missouri, Columbia, MO, United States

**Keywords:** water use efficiency, ^13^C/^12^C isotopic ratio, soybean, drought tolerance, association mapping, genomic selection, genomic estimated breeding values, quantitative

## Abstract

Soybean is a major legume crop cultivated globally due to the high quality and quantity of its seed protein and oil. However, drought stress is the most significant factor that decreases soybean yield, and more than 90% of US soybean acreage is dependent on rainfall. Water use efficiency (WUE) is positively correlated with the carbon isotopic ratio ^13^C/^12^C (C13 ratio) and selecting soybean varieties for high C13 ratio may enhance WUE and help improve tolerance to drought. Our study objective was to identify genetic loci associated with C13 ratio using a diverse set of 205 soybean maturity group IV accessions, and to examine the genomic prediction accuracy of C13 ratio across a range of environments. An accession panel was grown and assessed across seven distinct combinations of site, year and treatment, with five site-years under irrigation and two site-years under drought stress. Genome-wide association mapping (GWAM) analysis identified 103 significant single nucleotide polymorphisms (SNPs) representing 93 loci associated with alterations to C13 ratio. Out of these 93 loci, 62 loci coincided with previous studies, and 31 were novel. Regions tagged by 96 significant SNPs overlapped with 550 candidate genes involved in plant stress responses. These confirmed genomic loci could serve as a valuable resource for marker-assisted selection to enhance WUE and drought tolerance in soybean. This study also demonstrated that genomic prediction can accurately predict C13 ratio across different genotypes and environments and by examining only significant SNPs identified by GWAM analysis, higher prediction accuracies (*P* ≤ 0.05; 0.51 ≤ *r* ≤ 0.65) were observed. We generated genomic estimated breeding values for each genotype in the entire USDA-GRIN germplasm collection for which there was marker data. This information was used to identify the top ten extreme genotypes for each soybean maturity group, which could serve as valuable genetic and physiological resources for future breeding and physiological studies.

## 1 Introduction

Soybean (*Glycine max* [L.] Merr.) is a valuable legume crop that provides essential nutrients, such as protein, oil, and minerals for human and animal nutrition. In 2022, the world produced over 348 million tons of soybean across more than 133 million hectares of land (https://www.fao.org/faostat/en/#data/QCL/visualize). Drought is the most significant factor that affects soybean growth and production (Dogan et al., 2007), and can decrease seed yield and can cause financial losses for farmers (Sinclair et al., 2007; Wei et al., 2018; Poudel et al., 2023). Furthermore, changing climactic and precipitation patterns can affect soybean yields (Foyer et al., 2016) which have the potential to exacerbate drought losses (Osei et al., 2023).

Water Use Efficiency (WUE), a physiological trait associated with drought tolerance, is defined as the amount of dry matter (e.g. soybean shoot biomass) produced per unit of water transpired (Avramova et al., 2019). Genetic variation associated with WUE responses has been documented in numerous crops including soybean (Hufstetler et al., 2007; Ingwers et al., 2021), cowpea (*Vigna unguiculata* (L.) Walp.) (Ashok et al., 1999), peanut (*Arachis hypogea* L.) (Hubick et al., 1988), rice (*Oryza sativa* L.) (Cabuslay et al., 2002), wheat (*Triticum aestivum* L.) (Siahpoosh and Dehghanian, 2012), barley (*Hordeum vulgare* L.) (Hubick et al., 1988), and cotton (*Gossypium* spp.) (Fish and Earl, 2009). In general, WUE is under strong genetic control and exhibits high heritability, allowing for genetic variation among genotypes and environments (Dhanapal et al., 2015a; Kaler et al., 2017a, Kaler et al., 2018a; Bazzer et al., 2020a, Bazzer et al., 2020b; Chamarthi et al., 2023).

Improving WUE is essential for maintaining or increasing yield under conditions of water scarcity and projected changes in climate. Unfortunately, measuring direct yield responses to drought and/or true WUE is extremely laborious and expensive (Ismail and Hall, 1992). Hence, breeding programs have largely avoided measuring WUE for large numbers of genotypes under field conditions (Ismail and Hall, 1992). Because direct measurement of WUE responses in the field is difficult and does not scale well, researchers have identified morpho-physiological traits that can increase crop yields under water limited conditions. Several recent studies by our group have proposed the following traits as morpho-physiological traits of interest for selection to increase drought tolerance in soybean: High ^13^C/^12^C ratio (Kaler et al., 2017a; Chamarthi et al., 2023), slow canopy wilting (Kaler et al., 2017b; Steketee et al., 2020; Chamarthi et al., 2021), lower canopy temperatures (Kaler et al., 2018b; Bazzer and Purcell, 2020), rapid canopy coverage (Kaler et al., 2018c) and reduced N2 fixation sensitivity to drought (Dhanapal et al., 2015b; Bazzer et al., 2020c). These traits have been used in breeding programs to develop drought-tolerant cereal crop varieties in wheat, rice, and barley (Ludlow and Muchow, 1990; Richards, 1996; Richards et al., 2002; Babu et al., 2003; Lanceras et al., 2004), as well as for development of soybean drought tolerant germplasm (Carter et al., 2016; Ye et al., 2019; Kunert and Vorster, 2020; Manjarrez-Sandoval et al., 2020; Fallen et al., 2023). Simulation studies on soybean have shown that at least one morpho-physiological trait (slow canopy wilting) is predicted to increase overall soybean yields if beneficial alleles can be appropriately deployed (Sinclair et al., 2010).

Carbon isotope discrimination (Δ^13^C, ‰) in C3 plants is a factor of the differences in diffusion between ^13^CO_2_ and ^12^CO_2_ (a, ~4.4‰), discrimination against the heavier ^13^CO_2_ molecule by Rubisco (b, ~27‰), and the ratio of CO_2_ inside (C_i_) and outside of the leaf (C_a_) (Farquhar and Richards, 1984). The equation for Δ^13^C is:


(1) $$\Delta^{13}\text{C}=\text{a}+\left(\text{b}-\text{a}\right)*\left(1-{\text{C}}_{i}/{\text{C}}_{a}\right)$$

When considering instantaneous gas exchange in leaves, WUE can be expressed as:


(2) $$\text{WUE}=\left[{\text{C}}_{\text{a}}*\left(1-{\text{C}}_{\text{i}}/{\text{C}}_{\text{a}}\right)\right]/\left[1.6*\left({\text{e}}_{\text{i}}–{\text{e}}_{\text{a}}\right)\right]$$

where e_i_ and e_a_ refer to the water vapor concentrations inside the leaf and the surrounding atmosphere, respectively, and 1.6 is a constant accounting for the differences in diffusivity between the heavier CO_2_ molecule and H_2_O. Hence, Δ^13^C is inversely associated with WUE due to a common dependence upon C_i_/C_a_. That is, as C_i_/C_a_ decreases, Δ^13^C decreases and WUE increases (Farquhar and Richards, 1984). Isotopic fractionation may also be expressed as a ratio between ^13^C relative to ^12^C (C13 ratio, ‰), in which case there is a positive association between C13 ratio and WUE (Bazzer et al., 2020a).

A high C13 ratio (i.e., high WUE) can result from a high photosynthetic rate that decreases the CO_2_ concentration inside the leaf (C_i_, Equation 1) to low levels. Alternatively, high WUE may result from low stomatal conductance restricting transpiration that also results in a decreased C_i_. Constitutive low stomatal conductance expectantly results in high WUE, which may be an advantage under drought conditions. However, low stomatal conductance under conditions of plentiful soil moisture will limit photosynthesis and may ultimately limit crop yield (Condon et al., 2004). In many species, a high C13 ratio positively correlates with WUE, while high Δ13C, ‰ negatively correlates with WUE (Farquhar and Richards, 1984; Mininni et al., 2022). This makes it possible to use C13 ratio as an indirect selection criterion in breeding to improve WUE and potentially increase yield under drought conditions (Brugnoli et al., 2020).

High-throughput sequencing technologies and advanced phenotyping methods have enabled the use of modern genomic tools, such as genome-wide association mapping (GWAM) and genomic selection (GS) to investigate the genetic control of WUE (Blum, 2011; Kaler et al., 2017a, Kaler et al., 2018a; Ravelombola et al., 2021; Chamarthi et al., 2023). In soybean, the availability of various genomic marker tools and information, such as Simple Sequence Repeat Markers (Cregan, 2008), and the SoySNP50K iSelect SNP Beadchip from Illumina (Song et al., 2013; Song et al., 2015), has allowed the identification of significant genomic regions associated with various WUE-related traits like canopy wilting (Charlson et al., 2009; Hussein et al., 2012; Hwang et al., 2015, Hwang et al., 2016; Kaler et al., 2017b; Ye et al., 2019; Steketee et al., 2020; Chamarthi et al., 2021), canopy temperature (Kaler et al., 2018b; Bazzer and Purcell, 2020), and dark green color index (Kaler et al., 2020a).

The aim of this study is to discover novel loci associated with C13 ratio and to confirm loci previously identified through GWAM (Dhanapal et al., 2015a; Kaler et al., 2017a; Chamarthi et al., 2023) and/or linkage mapping (Bazzer et al., 2020a, Bazzer et al., 2020b) using a new panel of 205 diverse soybean maturity group (MG) IV accessions. We identified substantial overlap of QTL identified in this study with prior studies on drought-associated morphological traits, including oxygen isotope ratio (^18^O/^16^O, correlated with transpiration rate), canopy wilting, canopy temperature, nitrogen isotopic (^15^N/^14^N) ratio, nitrogen concentration, C/N ratio, and nitrogen derived from the atmosphere (Ndfa). Furthermore, we used this and prior datasets to test the accuracy of genomic prediction and applied to predict C13 ratio for >19,000 genotypes representing all maturity groups within the United States Department of Agriculture (USDA) soybean germplasm collection, in order to assist ongoing efforts to create more drought tolerant soybean cultivars.

## 2 Materials and methods

### 2.1 Plant materials

Chamarthi et al. (2021) detailed the selection of 205 MG IV soybean accessions used in this study, including 199 accessions and six check lines: PI 416937 (slow wilting), PI 471938 (slow wilting), A5959 (fast wilting), 08705_16 (fast wilting breeding line (Hwang et al., 2015)), a MG IV elite breeding line LG11-8169-007F (Gillen et al, 2018), and a non-nodulating check line ‘Lee non-nod’ (Bhangoo and Albritton, 1976). LG11-8169-007F and ‘Lee non-nod’ were planted in each of 12 incomplete blocks in each field using an augmented incomplete block design as controls for nitrogen fixation, which is not relevant to this study. The other 203 accessions were randomly assigned to an incomplete block within three replications per treatment/site. However, the data in this study was analyzed through a random complete block split-plot design. Out of the 205 accessions, 99 accessions were chosen because they collectively encompass the majority of genetic diversity present in a panel of 373 accessions used by Kaler et al. (2017b), while the remaining 100 accessions were selected from the USDA-GRIN collection (https://npgsweb.ars-grin.gov/) based on predicted extremes for C13 ratio (Dhanapal et al., 2015a; Kaler et al., 2017a), canopy wilting (Kaler et al., 2017b), N2 fixation (Dhanapal et al., 2015b), and canopy temperature (Kaler et al., 2018b) using breeding values (BVs) calculated from earlier association mapping studies.

### 2.2 Field sites and management

The experiment was designed with two treatments, irrigated (IR) and drought (DR), which were imposed in side-by-side field experiments at each of three different sites during the 2018 and 2019 cropping seasons. We will refer to the combination of site, year, and treatment as an “environment.” Research sites were the Bradford Research Center at Columbia (CO, 38.897 N, −92.2180), MO; the Pine Tree Research Station (PT, 35.2547 N, −90.7965), AR; and the Rohwer Research Station (RH, 33.8102 N, −91.2777), AR. However, no experiments were conducted in 2019 at the RH site, resulting in available data from only five site years across two treatments. Due to frequent rainfall during growing seasons these site years did not experience drought stress, and data from the PT 2018, RH 2018, and CO 2019 drought treatments could not be collected. Two of the seven site-years had both IR and DR treatment combinations, while three had only IR treatment. The site-year-treatment combinations for 2018 were designated as follows: IR (CO18IR) and DR (CO18DR) at Columbia, MO; IR (PT18IR) at Pine Tree, AR; and IR (RH18IR) at Rohwer, AR. For 2019, the designations were IR (CO19IR) at Columbia, MO; and IR (PT19IR) and DR (PT19DR) at Pine Tree, AR.

At the Columbia site, plots were 4-rows wide and 3.96 m long, with 34 seeds were planted per m^2^, and spacing between rows was 38 cm). The PT and RH locations were planted as 9 row plots sown using a drill with 19 cm between rows and plot lengths of 4.57 m. At the PT and RH sites, germination tests were used to adjust seeding to 32 m^-2^. At each site-year, phosphorus and potassium were applied based on soil test results to meet individual state recommendations, and pesticides were applied as needed for that specific site. Drip irrigation was used at the CO site, with irrigation carried out when soil moisture reached less than -30 kPa at 15 cm. Depth furrow irrigation was used at RH site, and flood irrigation at the PT site. For the PT and RH sites, both the IR and DR treatments received irrigation before the vegetative six (V6) stage when the estimated soil moisture shortage deficit exceeded 50 mm (Purcell et al., 2007). No further irrigation was applied at any site for the DR treatment after the V6 stage.

**Supplementary Table S1** provides details on each site’s latitude and longitude, number of rows per plot, plot length, plot width, type of irrigation, planting date, C13 sample collection dates, average maximum and minimum temperatures, total precipitation between planting and sample collection, number of irrigations, and cumulative potential evapotranspiration for the seven site-year-treatment combinations. To estimate the soil moisture deficit for the DR treatments, we calculated the cumulative potential evapotranspiration between emergence and plant sampling, as described by Purcell et al. (2007).

### 2.3 Phenotyping C13 ratio in plant tissues

Above ground material for five individual randomly selected plants from within all rows of each genotype replicate were collected between full bloom (R2) and beginning seed (R5) (Fehr et al., 1971). After harvest, the plant samples were dried at 60°C until a constant weight was achieved. Using a multistep grinding process (Dhanapal et al., 2015a), samples were packaged and sent to the University of California Davis Stable Isotope Facility for isotope analysis. Measuring absolute isotope composition is difficult; therefore, C13 ratio was expressed relatively to the international standard of the ^13^C/^12^C ratio Vienna PeeDee Belemnite (V-PDB). The website (https://stableisotopefacility.ucdavis.edu/) of the Stable Isotope facility contains additional information and details.

### 2.4 Statistical analyses

After consultation with statisticians at the University of Arkansas (Dr. Edward E. Gbur Jr. and Kevin C. Thompson), Analysis of variance (ANOVA) was performed using the PROC GLIMMIX procedure of SAS 9.4 (SAS Institute Inc. 2023. “The GLIMMIX Procedure” in *SAS/STAT^®^ 15.3 User’s Guide*. Cary, NC) to determine interactions among genotype, site year, and treatment. The statistical model for this analysis was: Y_ijkl_ = μ + G_i_ + S_j_ + T_k_ + GS_ij_ + GT_ik_ + ST_jk_ + GST_ijk_ + R_l (jk)_ + (residual error ϵ_ijkl_). In this model, fixed effects included G_i_ = the effect of the i^th^ genotype, S_j_ = the effect of the j^th^ site year, T_k_ = the effect of the k^th^ treatment, as well as all the fixed effect 2-way interactions (GS_ij_, GT_ik_, and ST_jk_), and the 3-way interaction GST_ijk_. The random effect is R_l (jk)_ = the effect of the l^th^ replicate nested in site year and treatment. We used ESTIMATE statement to generate the Best Linear Unbiased Estimates (BLUEs) for each genotype in three contexts: (1) for individual site-year-treatment combinations; (2) for each genotype across the two treatment combinations where data was available for both treatments; and (3) for each genotype across all site-year-treatment combinations (AAE). Raw Data is included in **Supplementary Table S2**. The BLUEs which were employed in GWAM and are provided for all genotypes in **Supplementary Table S3**.

To estimate the broad-sense heritability (*H* ^2^), we used the same PROC GLIMMIX procedure of SAS 9.4, applying the restricted maximum likelihood (REML) method to estimate variance components with all effects treated as random effects. Heritability was calculated using the following formulas:

where $\sigma_{G}^{2}\;$ is the genotypic variance, $\sigma_{GS}^{2}$ is the genotype by site year variance, $\sigma_{GST}^{2}$ is the genotype by site year by treatment variance, *k* is the number of site years, $\sigma_{\epsilon }^{2}$ is the residual variance, and *r* is the number of replications (Fehr, 1987; Bernardo, 2002). To assess consistency across environments, Pearson correlation coefficients were calculated using the ‘psych’ package in R (Revelle, 2017).

### 2.5 Genotypic data

We obtained marker data for 205 accessions in SoyBase (www.soybase.org) using the Illumina Infinium SoySNP50K iSelect SNP Beadchip previously collected (Song et al., 2013; Song et al., 2015), and the Glyma.w82.a1 genome assembly to ensure consistent positions as reported in previous GWAM of C13 ratio (Kaler et al., 2017a). Out of the 205 accessions, 201 had marker data (42,449 SNPs). These SNPs were subjected to quality control using TASSEL 5.0 (Bradbury et al., 2007) by removing SNPs with a minor allele frequency (MAF) ≤ 5%, SNPs with a missing rate higher than 10%, monomorphic, and all heterozygous SNPs were set to missing. After quality control, the final number of SNPs was 34,680. We imputed missing data that was ≤ 10% in the filtered SNPs using an LD-kNNi method (Money et al., 2015). We then used the filtered and imputed SNP set for GWAM to identify significant SNPs.

### 2.6 Genome-wide association mapping

We used the FarmCPU R package (Liu et al., 2016) to perform GWAM because it has been shown to effectively minimize the occurrence of false positives and false negatives (Kaler et al., 2020b). We identified significant SNPs using a threshold value of -Log10 *P* ≥ 3.5, equivalent to a *P* value ≤ 0.0003, which has previously been reported to be appropriate (Kaler and Purcell, 2019) and has been used in previous studies (Kaler et al., 2020a; Kaler and Purcell, 2020; Chamarthi et al., 2021; Chamarthi et al., 2023).

To identify common significant SNPs present in multiple site-year-treatment combinations, we used a *P* value threshold of ≤ 0.05, provided that the SNP was significant with a *P* value ≤ 0.0003 in at least one other environment (Kaler et al., 2017a; Kaler et al., 2017b; Kaler et al., 2020a; Chamarthi et al., 2021). We calculated allelic effects for each significant SNP by taking the mean difference in C13 ratio between the genotypes with the major and minor alleles. Both major and minor alleles were considered favorable if they were associated with an increased C13 ratio. A positive sign (+) in the allelic effect indicates that the major allele is associated with an increased C13 ratio. In contrast, a negative sign (-) indicates that the minor allele is associated with an increased C13 ratio.

In order to identify overlaps between our study and previous drought-related studies, we utilized the BEDtools Intersect Intervals Tool (Quinlan and Hall, 2010) in Galaxy (Community, 2024) with an overlapping QTL region of ± 175 kb. We chose this window size because the average LD across all chromosomes decayed to an average of 175 kb in the euchromatic region, as previously described (Chamarthi et al., 2021, Chamarthi et al., 2023). We considered SNPs that were not coincident with earlier studies as novel loci.

### 2.7 Genomic predictions and prediction accuracy for C13 ratio

Two datasets were used to compare GEBVs, produced through the BayesB genomic prediction model (Pérez et al., 2010). The first dataset consisted of a training population of 373 genotypes averaged over four site-years from a prior study (Kaler et al., 2017b). The testing population was the 201 genotypes drawn from the current study. The second dataset consisted of a training population of 201 genotypes used in the present study, with C13 BLUEs averaged over seven site-year-treatment combinations; the testing population was the 373 genotypes reported by Kaler et al. (2017b).

Two different marker subsets were used to evaluate the effects of marker distribution on prediction accuracy (Kaler et al., 2022). The first marker subset, SNP_All, included all 33,543 high-quality SNPs. The second marker subset, SNP_0.05, included only significant SNPs at *P* ≤ 0.05 (10,295) obtained from all site-year-treatment combinations. To calculate the prediction accuracy (correlation) of the C13 ratios, observed C13 ratio values were compared with predicted GEBVs obtained from the two different marker subsets (SNP_All and SNP_0.05) for the two different datasets. The R package (psych) was used to perform calculations (Revelle, 2017).

### 2.8 Predicting C13 ratio for soybean germplasm using GEBVs

The BayesB genomic prediction model was also utilized to predict the C13 ratios for the entire genotyped USDA-GRIN soybean germplasm collection for which marker data was available (Song et al., 2015), spanning Maturity Groups 000 to X. The prediction was based on a training population consisting of 201 genotypes from the present study’s C13 ratio BLUEs averaged over seven site-year-treatment combinations, while the testing population consisted of 19,285 genotypes of the USDA GRIN collection. The genotyping data of Glyma.w82.a1 was used to analyze the soybean germplasm sourced from Soybase (https://soybase.org/). GEBVs were generated for all accessions from each MG that had genotypic data, and the ten accessions with the highest and lowest C13 ratios were identified for each maturity group.

### 2.9 Candidate gene discovery

We examined regions defined by the significant SNPs discovered in the current study with soybean genes that have annotations (Glyma.w82.a1) which would suggest they have an involvement in abiotic stress responses. To do this, we used the Bedtools Intersect Intervals tool (Quinlan and Hall, 2010) and prepared two bed files. The first contained regions identified by centering each significant SNP identified in the current study within a window of ± 175 kb. This window size was used because the average LD across all chromosomes decayed at an average rate of 175 kb in the euchromatic region (Kaler et al., 2020a). The second bed file contained the soybean genome annotations (Glyma.w82.a1) obtained from SoyBase (https://soybase.org/). The output contained significant SNPs with overlapping candidate genes with their gene ontology (GO) categories (Chamarthi et al., 2021). Further, based on gene ontology (GO) biological function, we identified genes that were directly or indirectly associated with WUE and drought-related responses, such as abscisic acid, water deprivation, root development, leaf senescence, heat acclimation, and stomata (Schmutz et al., 2010; Sah et al., 2016).

## 3 Results

### 3.1 Phenotypic descriptions

There were substantial differences in the average maximum and minimum temperatures and total precipitation among seven different site-year-treatment combinations from emergence to the sampling dates (**Supplementary Table S1**). The average maximum temperature was highest at PT19 (32°C) and lowest at the CO18 (29°C) and CO19 (29°C) locations, whereas the average minimum temperature was highest in RH18 (23°C) and lowest in CO19 (12°C). The highest total precipitation was recorded in CO19 (457 mm) and the lowest in PT18 (305 mm). On the other hand, cumulative potential evapotranspiration was highest in CO18 (586 mm) and lowest in RH18 (414 mm).

**Figure 1:**
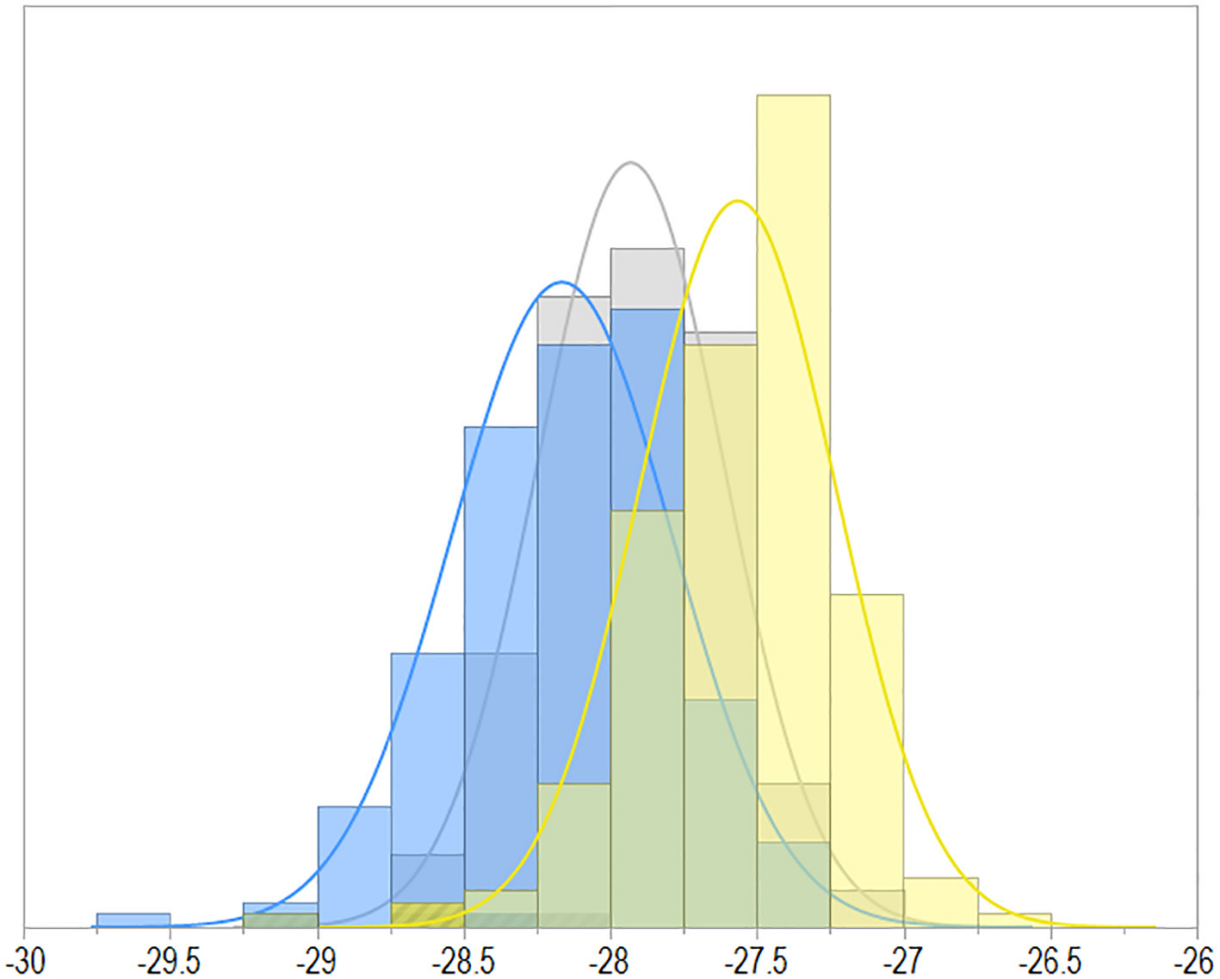
Frequency distribution of C13 ratio for 205 maturity group (MG) IV genotypic BLUEs averaged across two site-year-treatment combinations for both irrigated (blue) and drought treatments (yellow), and across all environment average (gray, AAE).

A descriptive analysis of C13 ratio revealed significant range across site years in the IR and DR treatments, including the Ave_2IR and Ave_2DR treatment combinations and AAE (**Table 1**; **Figure 1**). For the IR treatment, the minimum range of C13 ratio values was 1.81‰ (PT18IR), while the maximum range of C13 ratio values was 2.71‰ (PT19IR). For the DR treatment, the minimum range of C13 ratio values was 2.49‰ (CO18DR), and the maximum range of C13 ratio values was 2.76‰ (PT19DR) (**Table 1**). The frequency distribution of the average genotypic means of the C13 ratios for the Ave_2IR and Ave_2DR treatments indicated that there was a broad range of C13 ratios among both treatments (**Figure 1**). For the two site-years in which we had both IR and DR treatments, C13 ratio was less negative under drought as compared to irrigated treatment at both site-years (**Table 1**).

**Table 1:** Descriptive statistics and broad-sense heritability (H2) of C13 ratio for seven site-year-treatment combinations: Columbia (CO18 & CO19), Pine Tree (PT18 & 19), Rohwer (RH18) under irrigated (IR) and Columbia (CO18), Pine Tree (PT 19) under drought (DR) treatments, averaged over two irrigated (Ave_2IR), two drought (Ave_2DR) treatments, and averaged across all site-year-treatment combinations (AAE).

Environment	Mean ‰	Standard Deviation ‰	Range	*H^2^(%)*
CO18IR	-27.61	0.41	2.17	–
CO19IR	-27.93	0.39	2.26	–
PT18IR	-27.92	0.38	1.81	–
PT19IR	-28.69	0.41	2.71	–
RH18IR	-28.22	0.41	2.05	–
CO18DR	-26.75	0.40	2.49	–
PT19DR	-28.34	0.34	2.76	–
Ave_2IR	-28.16	0.38	2.27	73
Ave_2DR	-27.56	0.33	2.58	59
AAE	-27.93	0.32	2.10	90

**Figure 2:**
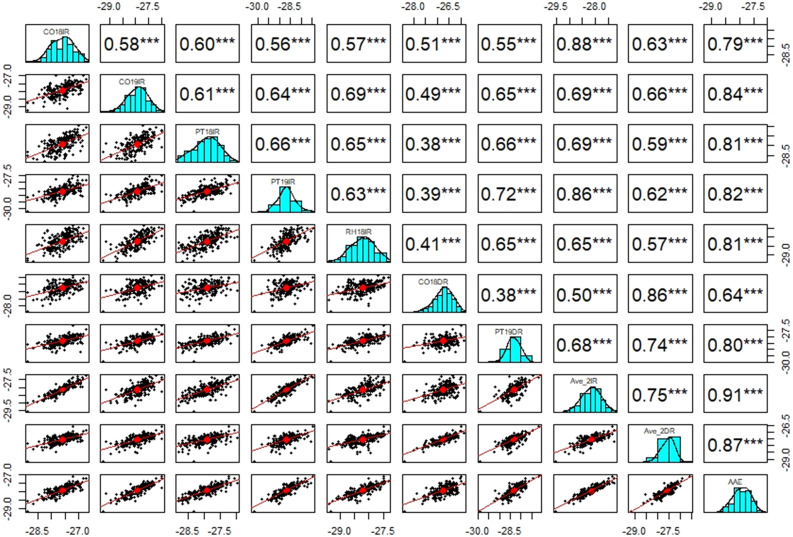
Correlations of C13 ratio (n=205) for Columbia (CO18 & CO19), Pine Tree (PT18 & 19), Rohwer (RH18) under irrigated (IR) and Columbia (CO18), Pine Tree (PT 19) under drought (DR) treatments and averaged over two irrigated (Ave_2IR) and two drought (Ave_2DR) treatments and averaged across site-year-treatment combinations (AAE). Significant at the *P* =< 0.0001^***^.

Analysis of variance across all seven site-year-treatment combinations demonstrated that the effects of genotype, site-year, treatment, and all two-way and three-way interactions were highly significant for C13 ratio (**Table 2**). For all site-year-treatment combinations, there were significant positive correlations (*P*< 0.0001) for C13 ratio between IR and DR treatments, and correlation between the Ave_2IR and Ave_2DR treatments was 0.75 (**Figure 2**). Broad-sense heritability (*H* ^2^) was 73.0%, 59.0% and 90% for the Ave_2IR treatments, for the Ave_2DR treatments, and across all environments (AAE) respectively (**Table 1**).

**Table 2:** Analysis of variance across site-year-treatment combinations for C13 ratio.

Effect	Degree of Freedom	F-statistic	P-value
Genotype (G)	204	15.48	<.0001
Site year (S)	4	469.28	<.0001
Treatment (T)	1	371.21	<.0001
G x S	808	1.62	<.0001
G x T	204	1.39	0.0003
E x T	1	65.64	<.0001
G x S x T	198	1.32	0.0027

Only fixed effects are shown.

### 3.2 Genome-wide association mapping

A total of 103 significant SNPs representing 93 loci associated with C13 ratio were found by GWAM (**Supplementary Figures S1**–**S3**, **Supplementary Tables S4**, **S5**). This includes 71 SNPs representing 62 loci that coincide with earlier studies investigating drought tolerance related traits including C13 ratio, canopy wilting, canopy temperature, N concentration, N isotope ratio, N derived from the atmosphere, C/N ratio, and O18 ratio (**Supplementary Table S4**). Another 32 SNPs representing 31 loci were found as novel loci (**Supplementary Table S5**).

Out of the 71 coincidental SNPs, a total of 41 SNPs representing 34 loci were identified from IR site-year-treatment combinations (on all chromosomes except Gm01, Gm10, Gm11 and Gm18). Another nine SNPs representing eight loci were identified from DR site-year-treatment combinations (on Gm01, Gm04, Gm09, Gm11, Gm14, Gm16, Gm17, and Gm20). For the Ave_2IR treatments, a total of eight SNPs representing eight loci from Ave_2DR treatments(on chromosomes Gm02, Gm06, Gm10, Gm14, Gm18, and Gm19) and five SNPs representing five loci were identified (on chromosomes Gm01, Gm02, Gm12, Gm14, and Gm15). Lastly, eight SNPs representing seven loci were found using our across all environments estimates (AAE, on chromosomes Gm06, Gm14, Gm18, Gm19, and Gm20, **Supplementary Table S4**). Of the 41 significant SNPs from the IR treatments, 28 were present in at least two site-year-treatment combinations. Of the nine significant SNPs from the DR treatments, eight were present in at least two site-year-treatment combinations. Interestingly, only five significant SNPs (ss715593828, ss715596390, ss715618057, ss715618082, and ss715635425) were identified in common between the IR and DR treatments, Ave_2IR treatments, Ave_2DR treatments, and AAE (on chromosomes Gm06, Gm07, Gm14, and Gm19, **Supplementary Table S4**). Allelic estimates were calculated for each significant SNP; a positive sign (+) indicates the major allele is associated with increased C13 ratio, whereas a negative sign (-) indicates the minor allele increased C13 ratio. In general, allelic effect estimates for individual loci were small, and ranged from -0.32 to 0.50‰ for the IR site-year-treatment combinations, -0.26 to 0.31‰ for the DR site-year-treatment combinations, 0.00 to 0.43‰ for the Ave_2IR treatments, -0.04 to 0.38‰ for the Ave_2DR treatments, and -0.01 to 0.42‰ for the AAE (**Supplementary Table S4**).

**Figure 3:**
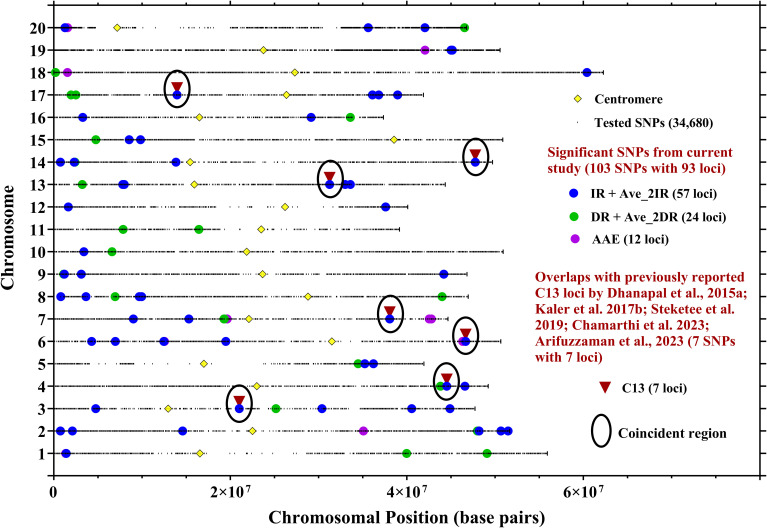
Locations of SNPs significantly associated with C13 ratio in seven site-year-treatment combinations, averaged over two IR treatments (Ave_2IR), averaged over two DR treatments (Ave_2DR), and averaged across site-year-treatment combinations (AAE). Locations of SNPs associated with C13 ratio from the current research study were compared with SNPs earlier identified with C13 ratio. Full details on overlaps are located in **Supplementary Table S4**.

Seven loci from IR site-year-treatment combinations (on chromosomes Gm03, Gm04, Gm07, Gm13, Gm14, and Gm17) as well as Gm06 from Ave_2IR treatments, were confirmed in coincident genomic regions. These SNPs correspond with previously reported GWAS-QTLs related to water use efficiency, specifically for the C13 ratio (**Supplementary Table S4**; **Figure 3**), highlighting their potential to enhance water use efficiency.

**Figure 4:**
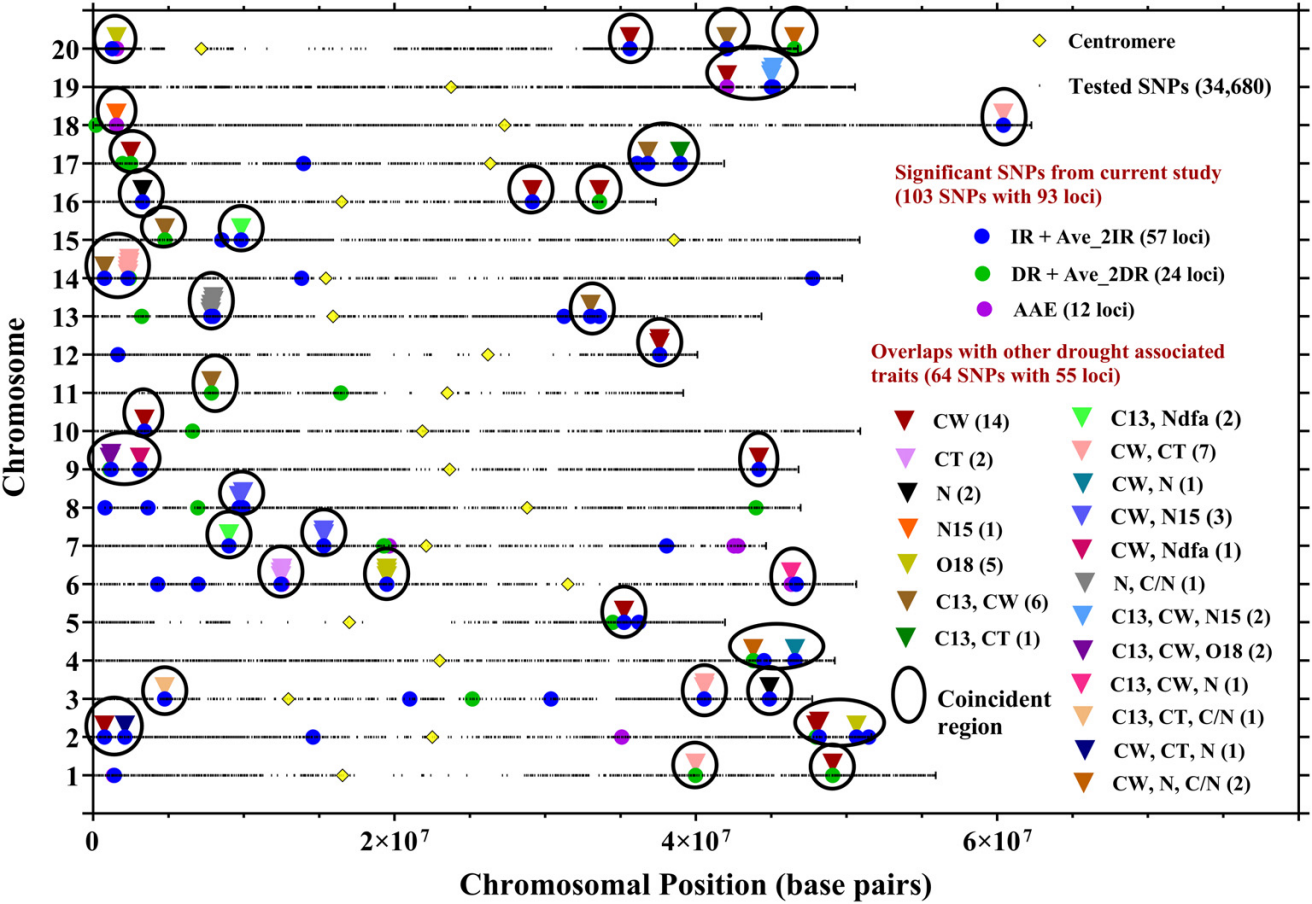
Locations of SNPs significantly associated with C13 ratio in seven site-year-treatment combinations, averaged over two IR treatments (Ave_2IR), averaged over two DR treatments (Ave_2DR), and averaged across site-year-treatment combinations (AAE). Locations of SNPs associated with C13 ratio from the current research study were compared with SNPs earlier identified with C13, O18, and N15 isotope ratios, nitrogen concentration (N), canopy wilt (CW), canopy temperature (CT), nitrogen derived from the atmosphere (Ndfa) and C/N ratio (C/N). Full details on overlaps are in **Supplementary Table S4**.

Additionally, due to the close relationship between water use efficiency (C13 ratio) and canopy wilting, six loci from IR treatments (on chromosomes Gm13, Gm17, and Gm20), Gm11 from DR treatment, Gm14 from Ave_2IR, and Gm15 from Ave_2DR treatments were confirmed in overlapping genomic regions. These loci are associated with both water use efficiency and canopy wilting traits (**Supplementary Table S4**; **Figure 4**). This indicates potential pleiotropic effects that could allow for simultaneous improvements in both water use efficiency and canopy wilting. Furthermore, other drought trait loci, such as canopy temperature, nitrogen isotope ratio, oxygen isotope ratio, and nitrogen derived from the atmosphere, overlap with C13 ratio loci (**Supplementary Table S4**; **Figure 4**). This further supports the hypothesis of pleiotropy among drought tolerance traits, which could facilitate genomic or marker-assisted selection.

We consider SNPs not coincidental with prior research as novel (**Supplementary Table S5**). Out of the 32 significant novel SNPs identified, 12 SNPs representing 12 loci from IR site-year-treatment combinations (were on chromosomes Gm02, Gm03, Gm05, Gm06, Gm12, Gm13, Gm14, Gm15, Gm17, and Gm20), seven SNPs representing six loci were from DR site-year-treatment combinations (on chromosomes Gm03, Gm07, Gm10, Gm17, and Gm18), three SNPs representing three loci were from Ave_2IR treatments (on chromosomes Gm01, and Gm08), five SNPs representing five loci were from Ave_2DR treatments (on chromosomes Gm05, Gm08, Gm11, and Gm13), and five SNPs representing five loci were from AAE (on chromosomes Gm01, Gm02, and Gm07). Of the 12 significant SNPs from the IR treatments, six SNPs were present in at least two site-year-treatment combinations. Of the seven significant SNPs from the DR treatments, two were present in at least two site-year-treatment combinations. There was one significant SNP (ss715626857) on chromosome Gm17 common to both IR and DR site-year-treatment combinations (**Supplementary Table S4**).

The allelic effects of these novel SNPs ranged from -0.40 to 0.47‰ in the IR site-year-treatment combinations, 0.00 to 0.41‰ in the DR site-year-treatment combinations, -0.07 to 0.15‰ in the Ave_2IR treatments, -0.17 to 0.21‰ in the Ave_2DR treatments, and -0.19 to 0.19‰ for the AAE (**Supplementary Table S4**). Several SNPs exhibited significant allelic effects and are located near genes involved in water transport, stomatal complex morphogenesis, root development, and root hair cell differentiation (**Supplementary Table S9**). For example, novel SNP ss715636628 on chromosome Gm20 displayed a positive allelic effect of 0.47‰, indicating that the major allele promotes water use efficiency (C13 ratio) in IR site-year-treatment combinations (**Supplementary Table S4**). This SNP is located near genes (*Glyma20g01760, Glyma20g01690, Glyma20g01610*, and *Glyma20g01895*) associated with biological functions relevant to water transport, heat response, lateral root development, root hair cell differentiation, and abscisic acid signaling (**Supplementary Table S9**). Similarly, novel SNP ss715591055 on chromosome Gm05 showed a positive allelic effect of 0.21‰, suggesting that the major allele also enhances water use efficiency (C13 ratio) in Ave_2DR treatments (**Supplementary Table S4**). This SNP is near genes (*Glyma05g28665, Glyma05g28610, Glyma05g28800*, and *Glyma05g28500*) related to lateral root morphogenesis, heat response, and jasmonic acid stimulation (**Supplementary Table S9**).

### 3.3 Identification of candidate genes for identified QTLs

Of the 103 total significant SNPs, seven SNPs had no genes with ontologies which matched our criteria within ± 175 kb, whereas 550 candidate genes were identified within ± 175 kb of 96 significant SNPs. Of these, 410 candidate genes were identified as coincident with SNPs previously identified as associated with C13 ratio and/or other drought tolerance physiological traits (as listed in **Supplementary Tables S4**, **S8**), while the remaining 140 were located in the vicinity of novel SNPs (as listed in **Supplementary Tables S4**, **S9**). Among the 96 SNPs, 10 were not just near but physically located within genes that encode proteins associated with plant stress responses. The candidate genes and their associated functions in root development, water transport, heat, cold, salt stress, auxin, abscisic acid, gibberellic acid, jasmonic acid, leaf senescence, and regulation of stomatal movement are all provided in **Supplementary Tables S8**, **S9**.

### 3.4 Genomic prediction of C13 ratio for soybean germplasm and accuracy

We evaluated two marker subsets to test the accuracy of genomic prediction for C13 ratio using two separate training and testing population combinations. Correlations were moderate and ranged from 0.30 to 0.65 (**Tables 3**, **4**). We also compared the performance of models using all available SNPs (SNP_All) with a subset consisting of only GWAM markers significant at *P* ≤ 0.05 (SNP_0.05). The results showed that SNP_0.05 had a higher accuracy than SNP_All (**Tables 3**, **4**). Prediction accuracies (*r*) were also higher when we used a larger training population of 373 genotypes, 0.59 and 0.65 (**Table 3**), as compared to the smaller training population of 201 genotypes which had *r* = 0.50 and 0.51 for SNP_All and SNP_0.05 respectively (**Table 4**).

**Table 3:** Prediction accuracy of C13 Genomic Estimated Breeding Values (GEBVs) with observed C13 ratios for seven site-year-treatment combinations in the current study.

Environment	Treatment	SNP_All	SNP_0.05
Columbia/2018	Irrigated	0.46***	0.56***
Columbia/2019	Irrigated	0.47***	0.53***
Pine Tree/2018	Irrigated	0.55***	0.60***
Pine Tree/2019	Irrigated	0.53***	0.53***
Rohwer/2018	Irrigated	0.59***	0.62***
Columbia/2018	Drought	0.35***	0.43***
Pine Tree/2019	Drought	0.45***	0.49***
Averaged	Two irrigated	0.50***	0.56***
Averaged	Two drought	0.40***	0.46***
Averaged	Across	0.59***	0.65***

Significant at the P =< 0.0001***,< 0.001**,< 0.05*. The genomic predictions were estimated using 373 genotypes as a training set and 201 genotypes as a testing set. SNP_All: Prediction accuracy with all SNPs (33,543) used in the training set to predict the GEBVs. SNP_0.05: Prediction accuracy with significant SNPs at *P* ≤ 0.05 (10,295) used in the training set to predict the GEBVs.

**Table 4:** Prediction accuracy of C13 Genomic Estimated Breeding Values (GEBVs) with observed C13 ratios for four site-year-treatment combinations reported by Kaler et al. (2017b).

Environment/Year	SNP_All	SNP_0.05
Columbia/2009	0.44***	0.44***
Columbia/2010	0.39***	0.40***
Stuttgart/2009	0.36***	0.30***
Stuttgart/2010	0.34***	0.41***
Averaged across environments	0.50***	0.51***

Significant at the P =< 0.0001***,< 0.001**,< 0.05*. The genomic predictions were estimated using 201 genotypes as a training set and 373 genotypes as a testing set. SNP_All: Prediction accuracy with all SNPs (33,543) used in the training set to predict the GEBVs. SNP_0.05: Prediction accuracy with significant SNPs at P ≤ 0.05 (10,295) used in the training set to predict the GEBVs.

We used AAE for C13 ratio BLUEs of 201 genotypes from the current study as a training set to predict C13 values for 19,285 soybean genotypes for which marker data was reported by Song et al. (2015). The genotypes showed a wide range of predicted C13 values, ranging from less than -29.00‰ to more than -27.00‰ (**Supplementary Figure S4**; **Supplementary Table S6**). **Supplementary Table S7** presents the ten genotypes with the highest and the ten with the lowest C13 ratio for each MG. The GEBVs for the ten accessions with the highest predicted C13 ratio ranged from -27.28 to -27.39‰ and were from MG IX. The GEBVs for the ten accessions with the lowest predicted C13 ratio ranged from -29.07 to -28.98‰ and were from MG VIII. The MG with the greatest range in GEBVs for C13 ratio was MG III (1.10), while the smallest range in GEBVs for C13 ratio was MG X (0.51).

## 4 Discussion

This research expands upon previous studies by verifying previously reported loci and identifying new loci that are linked to C13 ratio and/or other drought-related traits. These findings enhance our understanding of both the genetic control and environmental interactions that influence WUE in soybean.

### 4.1 Phenotypic descriptions

Environmental conditions varied considerably across the seven sites (**Supplementary Table S1**). Consistent with the literature, C13 ratio in general responded positively to limited water availability, leading to less negative C13 ratios particularly across non-irrigated site-year-treatment combinations (**Table 1**; **Figure 1**). This highlights the impact of environmental stress on isotopic composition and is consistent with previous studies. The high heritability (*H* ^2^) of C13 ratio (90% for AAE) in our study (**Table 1**) indicates strong genetic control and suggests that C13 ratio could be integrated as a selectable trait in breeding programs aimed at improving WUE in soybean (McCarthy et al., 2008). Moreover, the broad range of reactions among genotypes to diverse environmental conditions (G×E interactions) suggests the existence of genotype-specific adaptations that could be utilized to improve commercial soybean WUE, potentially without compromising crop productivity under varying water regimes (Chamarthi et al., 2023). We noted significant positive correlations (*P*< 0.0001, *r* = 0.75) between the phenotypic data from irrigated and drought treatments (IR and DR, respectively, **Figure 2**), which highlights consistent genetic responses to varying water availability conditions across different site-year-treatment combinations. Similar heritability and correlation results were found in previous mapping studies for C13 ratio (Dhanapal et al., 2015a; Kaler et al., 2017a; Bazzer et al., 2020a, Bazzer et al., 2020b).

### 4.2 Genome-wide association mapping

Previous studies in soybean have identified QTLs associated with traits that confer drought tolerance through both association and linkage mapping. These traits include C13 ratio (Dhanapal et al., 2015a; Kaler et al., 2017a; Steketee et al., 2019; Bazzer et al., 2020a, Bazzer et al., 2020b; Arifuzzaman et al., 2023), O18 ratio (Kaler et al., 2017a), canopy wilting (Charlson et al., 2009; Hussein et al., 2012; Hwang et al., 2015, Hwang et al., 2016; Kaler et al., 2017b; Steketee et al., 2020; Chamarthi et al., 2021; Li et al., 2023), canopy temperature (Kaler et al., 2018b; Bazzer and Purcell, 2020), Nitrogen concentration and N15 ratio (Dhanapal et al., 2015b; Steketee et al., 2019; Bazzer et al., 2020c; Arifuzzaman et al., 2023), Nitrogen derived from the atmosphere (Ndfa) (Dhanapal et al., 2015b) and C/N ratio (Dhanapal et al., 2015b).

The current study confirmed that 62 loci overlap with those previously linked to C13 isotopic ratios (**Figure 3**) and other drought-related traits identified in previous studies (**Figure 4**). Among these, seven loci were specifically associated with C13 ratio reported by Dhanapal et al. (2015a); Kaler et al. (2017b); Steketee et al. (2019); Chamarthi et al. (2023); Arifuzzaman et al. (2023) (**Supplementary Table S4**; **Figure 3**). The remining 55 loci were linked to both C13 ratio and various drought-related traits, including canopy wilt (CW), canopy temperature (CT), oxygen isotope ratio (O18), nitrogen isotope ratio (N15), nitrogen concentration (N), C/N ratio, nitrogen derived from the atmosphere (Ndfa) (**Supplementary Table S4**; **Figure 4**). We report that these coincident SNPs were located near genes associated with water transport, stress response, root hair elongation, abscisic acid pathways, abiotic stresses, and regulation of stomatal movement (**Supplementary Table S8**). However, we acknowledge that our list of candidate genes are merely coincident with the regions detected by GWAM, and will require experimental validation using structured populations; this remains to future work. Nevertheless, coincident genomic regions associated with multiple drought-related traits provide breeders with valuable insights into genetic factors underlying control of WUE. Breeders may in the future exploit these shared genetic regions to simultaneously improve multiple drought-related traits through breeding strategies that target specific loci associated with WUE and other relevant agronomic traits such as yield. While this observation highlights the possible interconnectedness of physiological responses to water stress in soybean and supports the concept of pleiotropy between genes/loci controlling multiple stress-responsive traits, it is also possible that there are multiple causative loci which are physically linked. Nevertheless, analysis of individual loci would require development of structured mapping or breeding populations, which is left to future work.

In addition, the current study identified 32 novel SNPs representing 31 loci associated with C13 ratio. Several of these novel SNPs had large allelic effects. For instance, SNP ss715601564 on chromosome Gm08 had an allelic effect of 0.15‰ (**Supplementary Table S5**) when averaged over the irrigated site-year-treatment combinations (Ave_2IR), accounting for 7% of the variation among genotypes for this treatment. This SNP is located near the genes *Glyma08g05140, Glyma08g05150, Glyma08g05165, Glyma08g05200, Glyma08g04920, Glyma08g05361*, and *Glyma08g05370*, which are associated with root development, regulation of stomatal movement, leaf senescence, jasmonic acid signaling, response to water deprivation and salt stress (**Supplementary Table S9**).

Similarly, SNP ss715591055 on chromosome Gm05 had an allelic effect of 0.21‰ (**Supplementary Table S5**) when averaged over the drought site-year-treatment combinations (Ave_2DR), accounting for 8% of the variation among genotypes for this treatment. This SNP is located near the genes *Glyma05g28665*, *Glyma05g28610*, *Glyma05g28800*, and *Glyma05g28500*, which are also involved in root development, regulation of stomatal movement, leaf senescence, jasmonic acid signaling, responses to water deprivation and salt stress (**Supplementary Table S9**).

Additionally, SNP ss715578484 on chromosome Gm01 had an allelic effect of 0.19‰ (**Supplementary Table S4**) when averaged across site-year-treatment combinations (AAE), accounting for 9% of the variation among genotypes for AAE. This SNP is located near the genes *Glyma01g01730*, *Glyma01g01830*, *Glyma01g01875*, *Glyma01g01890*, *Glyma01g01900*, *Glyma01g01920*, and *Glyma01g01780*, which are associated with response to various abiotic stress or hormone annotations (**Supplementary Table S9**). Novel SNPs with large allelic effects may provide breeders with additional genomic resources for enhancing WUE in soybean under drought stress. While we cannot at present conclude that genetic variation for any of these candidate genes is directly responsible for the phenotypic effects we observed, our results do provide a solid framework for further investigations which was not present *a priori*.

Simulation studies have predicted that the integration of slow canopy wilting (which is positively correlated with C13 ratio) would result in increased soybean seed yield overall (Sinclair et al., 2010). As previously mentioned a high C13 ratio (i.e., high WUE) can result from a high photosynthetic rate that decreases leaf CO_2_ concentration levels (C_i_, Equation 1) or from low stomatal conductance, restricting transpiration. It is likely that both processes are being detected in our analyses. We acknowledge that when working with novel genetic material there is always the possibility for unintended negative impacts on seed yield (i.e. yield drag) or upon other important agronomic traits.

Hypothetically, yield drag or negative agronomic trait interaction could either be either due to direct or indirect factors. For example, a direct negative impact could be constitutive restriction of stomatal opening, which would result in less negative C13 ratios but would also limit evapotranspiration, which could decrease seed yield. Alternately, beneficial alleles for C13 ratio could be in linkage with other gene variants which could have undesirable negative effects on yield or agronomic traits. In the second case, the benefits of altering C13 ratio could be recouped if that linkage could be broken. One classical example of undesirable linkage in soybean was the tight 0.35 cM linkage (Matson and Williams, 1965; Lewers et al., 2002) between the *i* allele of the *Inhibitor* seed coat coloration gene (black seed coats are undesirable) and the soybean cyst nematode resistance gene *Rhg4* from black seeded line ‘Peking’. It was only through extensive backcrossing that this linkage could be broken (Brim and Ross, 1966). Today resistance alleles from *Rhg4* have been widely deployed and are essential to SCN resistance in thousands of released cultivars. Another example of yield drag is the recently identified *rag2* aphid resistance locus which has been determined, through genetic mapping and analysis of breeding populations, to cause an estimated −125 kg ha^−1^ effect on seed yield (Ward et al., 2017). It remains to be seed if this is due to direct pleiotropic effects of the *rag2* locus or due to linkage between two genetic loci, one which affects yield and another affecting resistance to aphids.

### 4.3 Genomic prediction accuracy

The accuracy of genomic selection in predicting GEBVs greatly depends on the size of the training population (VanRaden et al., 2009). Moreover, Kaler et al. (2022) reported that prediction accuracy also improves when only markers that are significantly (*P* ≤ 0.05) associated with a trait are used, rather than all genotyped markers. Our results confirmed that using significant SNPs (SNP_0.05) and a larger training population resulted in higher predictive power (*r* = 0.59 to *r* = 0.65, **Table 3**). This consistency in methodology strengthens the reliability and applicability of genomic prediction models across different environmental contexts.

### 4.4 Predicting C13 values for soybean germplasm

The prediction of C13 values for diverse soybean genotypes using C13 ratio BLUEs helps to understand isotopic composition. The broad range of predicted C13 values across maturity groups highlights the genetic diversity in soybean germplasm (**Supplementary Figure S4**; **Supplementary Table S6**), which is essential for breeding programs that target carbon isotopic composition for drought tolerance improvement. By using GEBVs, we identified soybean genotypes expected to exhibit extreme C13 ratios (**Supplementary Table S7**), which can serve as additional genetic resources for breeders to assist in selecting parental lines for future breeding efforts to increase WUE and physiologists and geneticists working to understand soybean drought tolerance mechanisms.

## 5 Conclusions

Our study has not only confirmed previous research but also provided new genetic resources for breeders to enhance WUE and drought tolerance in soybean. Our GWAM study has confirmed 62 loci related to drought-related traits and also identified another 31 novel loci associated with alterations to C13 ratio. The overlapping genomic regions associated with multiple drought-related traits have provided breeders with a better understanding of the genetic factors underlying WUE, enabling them to develop breeding approaches that target specific loci and improve multiple agronomic traits simultaneously. Our research has also found that incorporating only significant SNPs in genomic selection models improved the accuracy of predicting C13 ratio. Lastly, we have used genomic selection to predict C13 ratio for USDA soybean germplasm and found a wide range of predicted values across different maturity groups, providing valuable information for breeders to select parental lines in future breeding efforts.

The results of this study can be useful for enhancing soybean WUE through selective breeding. Future research could validate the key candidate genes identified in this study through functional genomics approaches and further explore their roles in mediating soybean’s response to stress and isotopic composition. Truly understanding the genetic basis of C13 ratio and how it responds to environmental factors will require future work but could lead to the development of soybean varieties more resilient to drought stress and changes in climate.

## Data Availability

The original contributions presented in the study are included in the article/**Supplementary Material**, further inquiries can be directed to the corresponding author/s.
